# Controls on fracture openness and reactivation in Forsmark, Sweden

**DOI:** 10.1038/s41598-023-33619-9

**Published:** 2023-04-24

**Authors:** D. Doolaeghe, C. Darcel, J.-O. Selroos, D. Mas Ivars, P. Davy

**Affiliations:** 1 University of Rennes, CNRS, Géosciences Rennes, UMR 6118, 263 Avenue General Leclerc, 35042 Rennes, France; 2 Itasca Consultants S.A.S., 29 Avenue Joannes Masset, 69009 Lyon, France; 3 Swedish Nuclear Fuel and Waste Management Co (SKB), Evenemangsgatan 13, Box 3091, 169 03 Solna, Sweden; 4 Department of Civil and Architectural Engineering, Division of Soil and Rock Mechanics, KTH Royal Institute of Technology, Brinellvägen 23, 100 44 Stockholm, Sweden; 5 Department of Sustainable Development, Environmental Science and Engineering, KTH Royal Institute of Technology, 100 44 Stockholm, Sweden

**Keywords:** Hydrology, Solid Earth sciences

## Abstract

In crystalline bedrock, the open fraction of the fracture network constitutes the main pathways for fluids. Many observations point out that the state of stress influences the open fraction, likely indicating recent reactivation. But how this occurs is still unresolved. We analyse the conditions for fracture reactivation from fracture data collected in the uppermost 1 km of bedrock in Forsmark, Sweden. The open fraction is mainly correlated to the stress acting normally on the fracture but even away from critical failure, leading us to analyse the potential fluid pressure required for reactivation, ${P}_{c}$. We observe that 100% of the fractures are open when ${P}_{c}$ is hydrostatic, and the ratio decreases exponentially to a plateau of ~ 17% when ${P}_{c}$ is lithostatic and above. Exceptions are the oldest fractures, having a low open fraction independent of ${P}_{c}$. We suggest that these results reflect past pressure build-ups, potentially related to recent glaciations, and developing only if the preexisting open fraction is large enough.

## Introduction

The state of fracture networks in crystalline bedrock generally results from a long deformation history, where the rock permeability and mechanical properties evolved due to successions of fracture failure and sealing processes^1–4^. Because fractures are the main conduits for flow and transport, understanding how these past events have shaped the current open fraction of the fracture network (called “openness” hereafter) is key to understanding geological reservoirs in the context of industrial applications, such as geothermal energy, carbon sequestration, or energy and waste storage projects. The present study focuses on the Forsmark site in Sweden. Forsmark was chosen to host a future deep repository for nuclear waste disposal and has a data-rich core-log database on fracture properties that is made available by SKB, the Swedish Nuclear Fuel and Waste Management company. Among all the fracture parameters examined in the current hydraulic models, like the size and the aperture distribution^5–8^, the spatial distribution of the openness remains rarely evaluated to date, even though it likely has a major impact on the network connectivity.

Records from Forsmark bedrock coring show that only a small part of the intersecting fractures is open ($\sim 20$%), reflecting either partial mineralisation of fractures and/or reopening of fractures resulting from mechanical reactivation. Forsmark bedrock has a long geological history with several episodes of deformations and fluid circulations, most of them older than ~ 1 Ga (Gothian orogeny, $1.7-1.5$ Ga; the Sveconorvegian orogeny, $1$ Ga; the far Caledonian orogeny, $500-400$ Ma). Loading and unloading cycles also occurred from sedimentary layers evolution and glaciation/deglaciation, for example, the numerous Quaternary glaciations^9^. The chemical activity is now negligible, and essentially no sealing is anticipated, except for occasional deposition in the very shallow transmissive fractures^9–11^. In a recent study, Moon et al.^12^ analysed how the current regional stress state can influence the fracture openness in Forsmark. They showed significant correlations with both tensile and shear stress mechanisms, from the surface to relatively large depths (~ 500 m). These correlations were increased by accounting for hydrostatic fluid pressure in the effective stresses. These results shed light on the role played by fracture reactivation on the current openness, but also raise the question of how this reactivation occurred, in particular at depth.

According to failure models, fracture reactivation can occur either by shearing when fractures are critically stressed or by tensile opening^13,14^. The permeability of fractured rocks has often been related to the presence of critically stressed fractures^15,16^. In Forsmark, the data show that very few fractures are currently in a critical state and that many open fractures are far from failure conditions. Tensile failure is expected close to the surface due to an increase of the ratio between horizontal and vertical stresses in the first few hundred meters (the ‘Brown–Hoek’ effect)^17,18^, but it does not address the problem of deep fractures for which the confining stress is high. If it is known how normal stress affects fracture aperture and transmissivity^19,20^, it is less clear how this mechanism causes the failure of sealed fractures.

Our goal is to characterize which mechanism has reactivated the fractures and led to the present fracture openness. In the continuity of the work of Moon et al.^12^, we first analyse how the openness, denoted ${f}_{op}$ (see “Openness calculation”), evolves with different stress indicators: the normal stress ${\sigma }_{n}$ and the distance to shear failure $\tau /{\tau }_{c}$(where $\tau$ is the shear stress and ${\tau }_{c}$ the critical shear stress). Our method consists of analysing these indicators in many fracture sets. These are selected by orientations, depths, structural domains (either fracture domains or deformation zones^9,21^), mineral fillings, and boreholes. We then examine the role of fluid pressure in fracture reactivation. Fluid pressure lowers the effective normal stress and could bring fractures close to failure envelopes. Hydrostatic fluid pressure (the weight of the water column) is commonly used, but it can be greatly exceeded if fluid cannot flow efficiently through the fracture network^22,23^. We define another indicator, ${P}_{c}$, which measures the potential fluid pressure required for the fracture to reach failure envelopes, and we perform the same analyses as for the previous stress indicators. In Forsmark, it has been suggested that pressure build-up occurred during the repeated glacial-interglacial episodes, allowing enhanced fluid diffusion and possible failure beneath the glacier front^24–27^. Even though the aforementioned studies indicate that the depth of such phenomenon is still under debate, we test this hypothesis by analysing the correlation between the openness and the critical pressure ${P}_{c}$.

## The sampled fracture set

Fractures observed in cores are any type of brittle discontinuity in rock, regardless of the failure mechanism, including also fractures that can have been sealed afterward by mineral precipitation. Ductile discontinuities such as dikes, sills, or pegmatite veins, are not considered. In Forsmark, fractures form a complex multiscale network of joints and faults that can be observed on surface outcrops (Supplementary materials: Fig. 13). The fraction of fractures recorded in the database depends on the recording criteria in the cores (only traces covering the centerline of the borehole are retained), the fracture size distribution (fsd), and the probability to intersect the boreholes, which is proportional to the square of the fracture size^28^. The fsd has been inferred from outcrop mapping^29,30^; it varies from one fracture domain to another, as the fracture density. Using fsd models constrained by outcrop trace distribution and enriched by fracture growth models^29^, we estimate that the fractures in the core database are mostly joints ranging in size from 10 cm to 1 km, with an average size between 1.5 and 5 m depending on the fracture density. They are thus likely to be similar to the fractures mapped on outcrops, which present a high percentage of T-intersections (one fracture abutting another)^31^.

Most of the fractures are either vertical or horizontal. If the density of vertical fractures is almost independent of depth, the density of horizontal fractures increases significantly in the first 100 m (see Darcel et al.^30^ and Supplementary materials: Figs. 7a, 8a), certainly in response to topographic, exhumation-related or thermal stresses^18,32^. Observing this increase in relation to the present surface, while the rocks have undergone numerous tectonic, burial, and exhumation phases in the past, seems to show that a significant portion of the horizontal fractures formed with recent conditions.

The mechanisms of fracture creation, which are mostly joints, are not the subject of this paper (see Martel^32^, Pollard and Aydin^33^). We only point out that the sampled fractures have a size much larger than those of microcracks likely induced by the relaxation of exhumation-related thermoelastic stresses^18,34^.

Most open fractures in the cores are filled with minerals, probably indicating the reactivation of previously sealed fractures (Supplementary Materials: Fig. 12). Only a few have no mineral at all (3,7% of the open fractures) and may represent recent fresh fractures^35^. It is not possible to constrain the spatial distribution of open surfaces^36^. Openness gives an estimate of the total percentage of open surface for a set of fractures.

## Results

### Dependency of the openness on fracture stress at Forsmark

**Figure 1:**
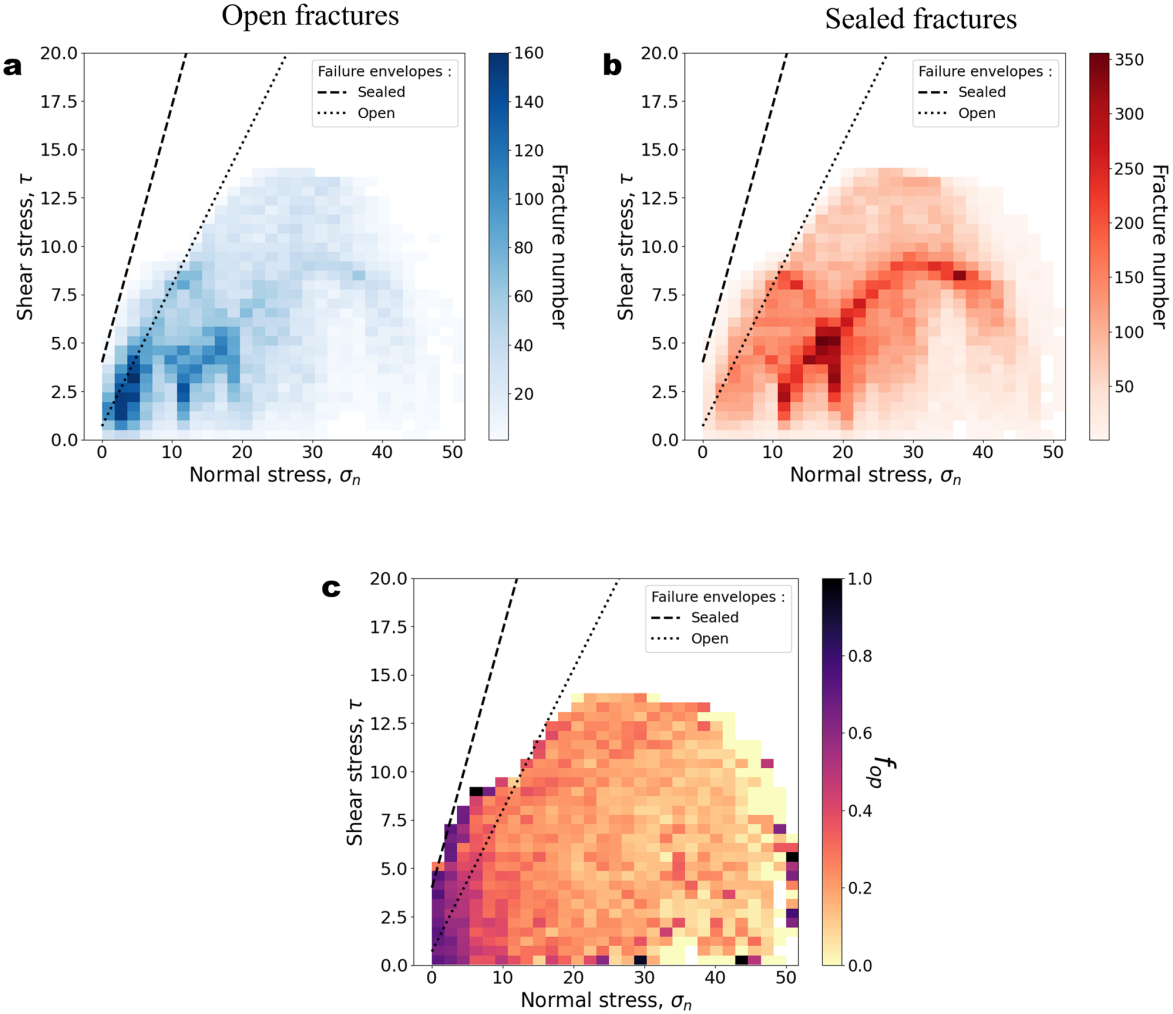
Fracture shear stress $\uptau (\mathrm{MPa})$ as a function of fracture normal stress ${\upsigma }_{\mathrm{n}} (\mathrm{MPa})$. (**a**) Open fractures. (**b**) Sealed fractures. (**c**) Fracture openness. $\tau$ and ${\sigma }_{n}$ are computed from Forsmark’s site stress model ^38^. The dashed and dotted lines indicate Mohr–Coulomb failure envelopes of sealed and open fractures, respectively, with parameters of friction $\mu$ and cohesion $c$ retrieved from loading tests performed on Forsmark samples (sealed fractures: $\mu =$ 1.33, $c=4$ MPa; open fractures: $\mu = 0.73, c=0.7$ MPa)^39^.

The data in the database containing the fracture characteristics was collected by SKB at Forsmark. It contains nearly 90,604 fractures collected along 40 boreholes with their characteristics measured in the wells and on the cores: depth, mineral fillings^10,37^, orientation, structural domain (either fracture domain or deformation zone defined primarily by the intensity of fracturing) and openness state (open, sealed and partly-open) (see “Dataset description”). We added stress characteristics by calculating the normal and shear projections (${\sigma }_{n}$ and $\tau$) of the stress tensor model established in Forsmark^38^ (see “Normal stress σ_n_ and shear stress τ in the fracture” and Supplementary Materials: Fig. 3). The failure envelope of existing fractures has been measured from tilt and shear tests on rock samples^39^. Our reference for estimating fracture opening by mechanical reactivation is the failure envelope measured for sealed fractures, which is a Mohr–Coulomb envelope with average cohesion $c$ of 4 MPa and friction coefficient $\mu$ of 1.33 (dashed line in Fig. 1). By comparison, the failure envelope for open fractures is indicated by the dotted line ($c=0.7$ MPa, $\mu =0.73$).

**Figure 2:**
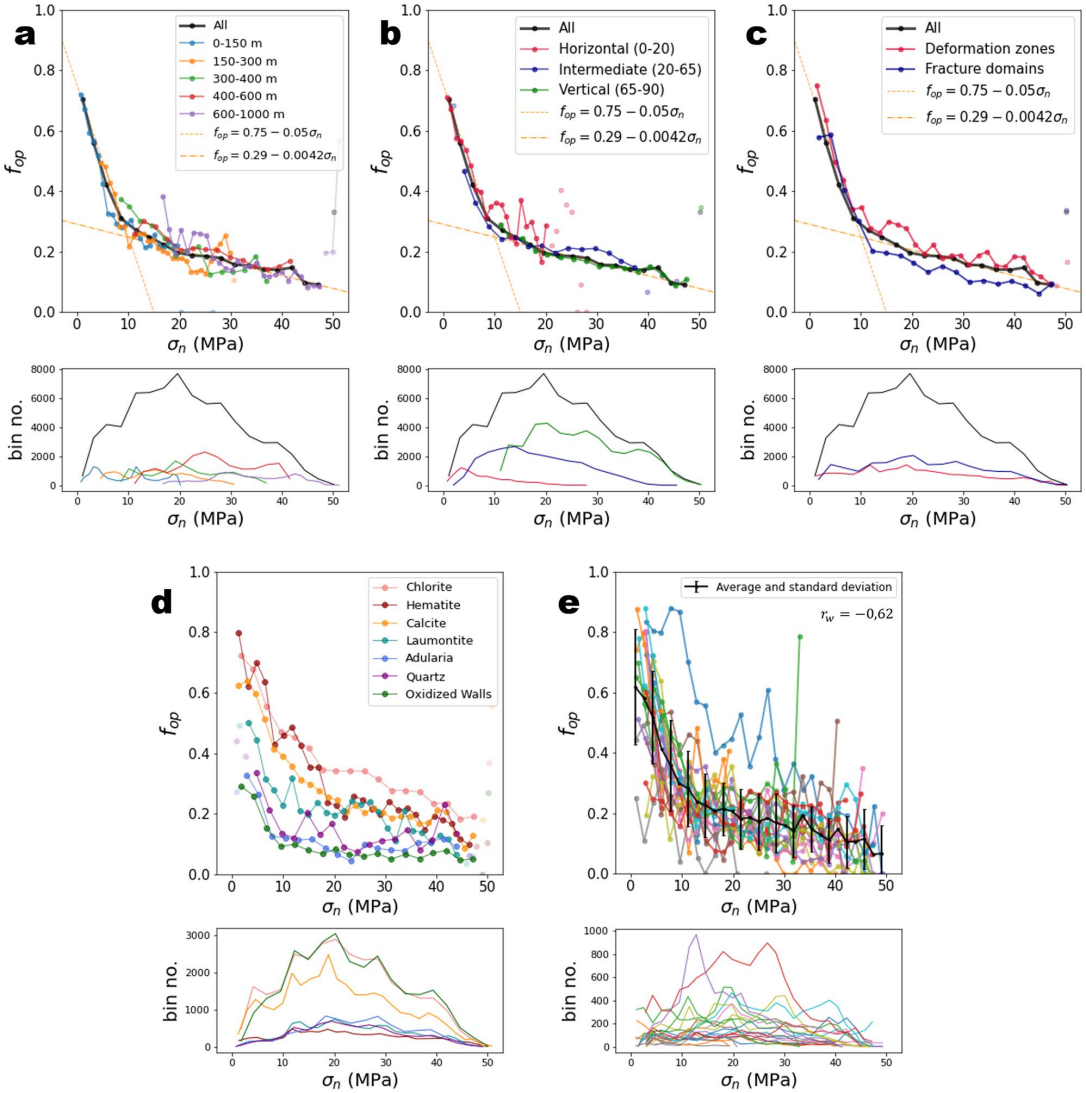
Openness ${f}_{op}$ as a function of fracture normal stress ${\sigma }_{n}$. The results are presented as a function of (**a**) depth range, (**b**) dip range, (**c**) structural domains, (**d**) filling minerals, and (**e**) by borehole. Transparent dots correspond to bins with fewer than 100 fractures. Lower subplots indicate the number of fractures in each bin. In each plot, bin size is 2 MPa. In (**e**), we calculated the Pearson correlation coefficient, weighted by the fracture number in each bin, ${r}_{w}$.

**Figure 3:**
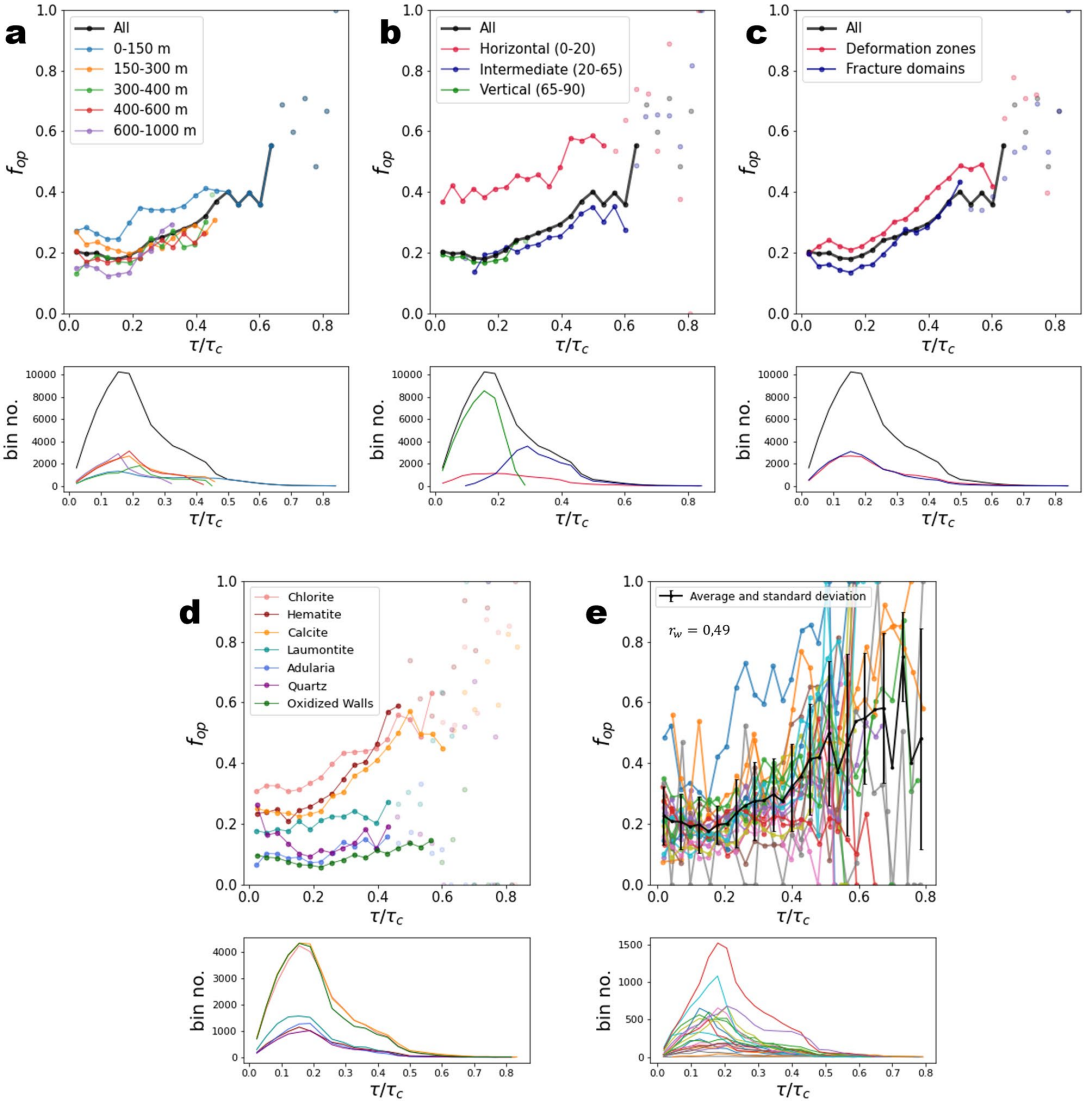
Openness ${f}_{op}$ as a function of the ratio $\tau /{\tau }_{c}$. The critical shear stress ${\tau }_{c}$ is calculated as ${\tau }_{c}=\mu .{\sigma }_{n}+c$, with $c=4$ MPa and $\mu =1.33$, cohesion and friction coefficients of sealed fractures. The results are presented as a function of (**a**) depth range, (**b**) dip range, (**c**) structural domains, (**d**) filling minerals, and (**e**) by borehole. Transparent dots correspond to bins with fewer than 100 fractures. Lower subplots indicate the number of fractures in each bin. In each plot, bin size is 0.033 MPa. In (**e**), we calculated the Pearson correlation coefficient, weighted by the fracture number in each bin, ${r}_{w}$.

No significant change in fracture openness state can be directly observed near the failure envelope. Both open and sealed fractures have stress characteristics well below the sealed-fracture failure envelope (Fig. 1a,b). The openness calculated in bins of ${\sigma }_{n}$ and $\tau$ likely presents a negative trend from low to high ${\sigma }_{n}$ (Fig. 1c). We further analyse these trends by calculating the openness as a function of the normal stress ${\sigma }_{n}$ and the distance to the sealed-fracture failure envelope in terms of shear stress, measured with the proxy, $\tau /{\tau }_{c}$ (Figs. 2 and 3). ${\tau }_{c}$ is the critical shear stress, calculated as ${\tau }_{c}=c+\mu *{\sigma }_{n}$.

The openness decreases when ${\sigma }_{n}$ increases with a strong variation before ~$10$ MPa and a smaller but still significant variation beyond (Fig. 2). This trend is almost independent of fracture orientation, depth, and structural domains (Fig. 2a–c), but it varies with the fracture-filling mineral (Fig. 2d). The spatial variability of the relationship is evaluated by calculating ${f}_{op}({\sigma }_{n})$ in each borehole (Fig. 2e). The standard deviation of ${f}_{op}$ computed by bin (vertical error bars) equals on average 0.1. The weighted Pearson correlation coefficient ${r}_{w}$ (“Weighted Pearson correlation coefficient *r*_*w*_”), calculated on the results by boreholes, indicates a significant negative correlation (${r}_{w}=-\mathrm{0,62}$). Selecting the fractures with large normal stress $({\sigma }_{n} >10 MPa)$, the correlation is less strong but still important (${r}_{w}=-\mathrm{0,47}$, see Table 1 in Supplementary Materials).

The openness increases with $\tau /{\tau }_{c}$ as expected—the closer to critical failure a fracture is, the larger the probability that it is open (Fig. 3). However, significant differences are observed between the depth ranges, the orientation groups or the structural domains (Fig. 3a–c), indicating that $\tau /{\tau }_{c}$ is not the right metric that controls the openness. In particular, the horizontal group of fractures (dip < 20°) do not have the same trend as the one measured for other fractures (Fig. 3b). The calculations performed by borehole (Fig. 3e) present a slightly larger variability and less significant correlation (${r}_{w}=0.49$) than the analysis with ${\sigma }_{n}$.

In the study of Moon et al.^12^, significant correlations were highlighted between the openness and both shear and tensile indicators. The present results show instead a control of ${\sigma }_{n}$, generalizable to all fractures, rather than the shear stress indicator, $\tau /{\tau }_{c}$. In addition, if a correlation is measured between the openness and the shear stress indicator, it must be, to some extent, related to the correlation measured with ${\sigma }_{n},$ as ${\tau }_{c}$ is function of ${\sigma }_{n}$.

Moon et al.^12^ also provided evidence that the correlations hold from the surface to depths of 500 m. Here, we complete this observation by showing that the correlation is the strongest for fractures with ${\sigma }_{n}<10$ Mpa, and weaker but still significant for fractures with ${\sigma }_{n}>10$ MPa. Note that this limit does not correspond to a precise orientation or depth as indicated by the overlaps between the different fracture groups of depth and orientation in Figs. 2a and b. In addition, we observe that the correlation likely still holds for the group of fractures from 600 to 1000 m (Fig. 2a, purple line) which is deeper than what is shown by Moon et al.^12^.

### Testing the hypothesis of a fracture reactivation by fluid overpressure

**Figure 4:**
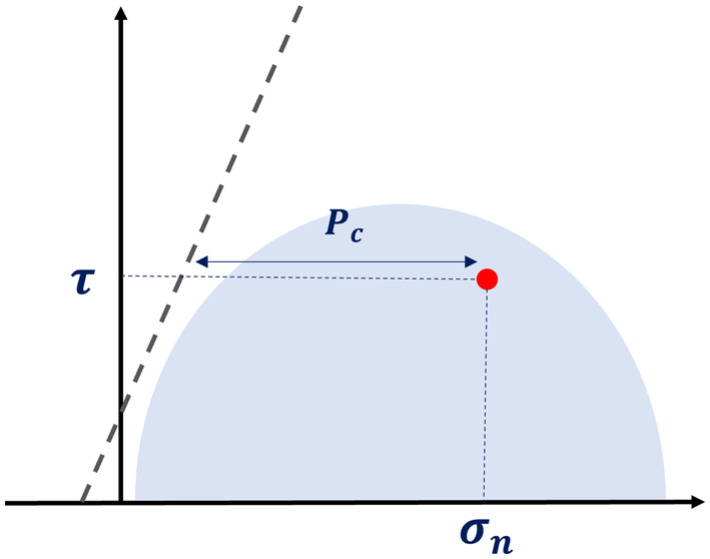
Schematic figure representing the critical fluid pressure ${P}_{c}$ (MPa) in a Mohr–Coulomb diagram. The red dot indicates a hypothetical fracture with ${\sigma }_{n}$ and $\tau$ estimated from the remote stress field.

We explore the possibility that openness is partly induced by fluid overpressure ${P}_{c}$, which takes the fracture closer to the failure envelope by reducing the effective stresses (Fig. 4). This process was originally invoked by Sibson to explain why faults remain seismically active while unfavourably oriented for fracture reactivation^22,23,40–43^. Fluid overpressures imply a complete or partial hydraulic disconnection of the deep zones from the surface, either permanent (e.g., low-permeability cap rock^41^ or disconnected fracture clusters) or transient (e.g., permeability decrease by fracture healing^23,44^), or a source of overpressure (e.g., ice cap^27,45–47^), or dehydration reactions^48^).

In the absence of fluid sources, the lithostatic pressure is generally considered as the upper limit of the overpressure for disconnected fractures^22,23,49,50^, but formally this limit depends on the applied stress, rock properties^51^ and the possibility of fracture clusters that span several depths. Noting that both hydrostatic and lithostatic pressures are reference values for overpressures, we build a metric ${P}_{c}^{*}$ of the fracture strength to failure by overpressure by (1) calculating the potential pressure ${P}_{c}$ required to reach the failure envelope for a fracture with a normal stress ${\sigma }_{n}$ and a shear stress $\tau$ (Fig. 4), and (2) normalising ${P}_{c}$ with both the hydrostatic (${P}_{H}$) and lithostatic (${P}_{L}$) pressure limits: ${P}_{c}^{*}=({P}_{c}-{P}_{H})/({P}_{L}-{P}_{H})$ (see “Fluid pressure analyses”). ${P}_{c}^{*}$, hereafter called the failure overpressure metric, is 0 for hydrostatic conditions and 1 for lithostatic conditions irrespective of the fracture depth, making it possible to compare fractures at different levels of the site. The failure envelope is that of sealed fractures, and the lithostatic pressure is calculated as the pressure term of the remote stress tensor, that is, the average of the tensor trace. Note that if we instead use the overburden pressure ${\sigma }_{v}$, which is generally taken as the lithostatic pressure^49^, ${P}_{c}^{*}$ is greater than or equal to 1, which means that ${\sigma }_{v}$ is barely enough to reactivate fractures (Supplementary Materials: Fig. 11).

**Figure 5:**
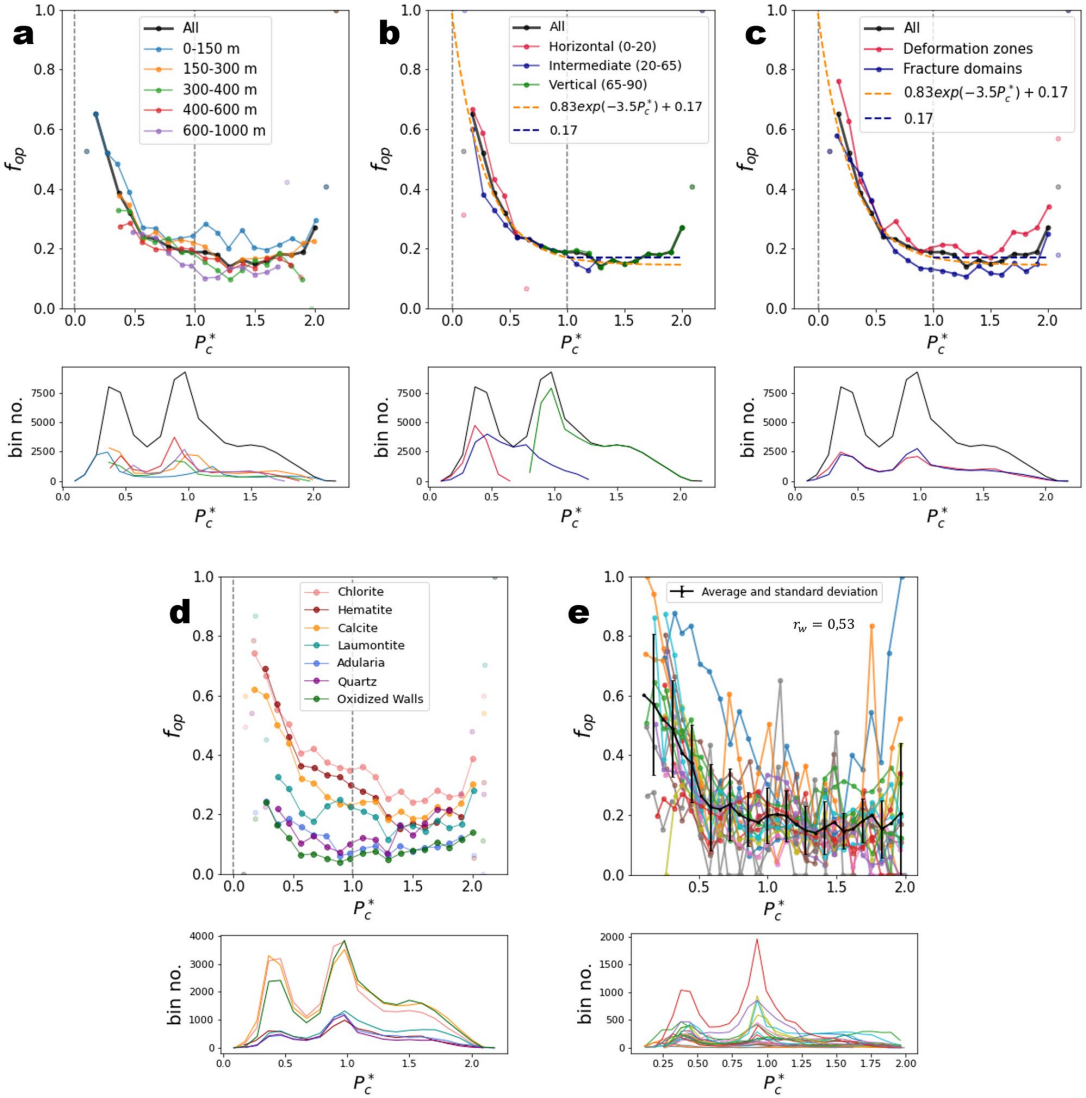
Openness as a function of the failure overpressure metric ${P}_{c}^{*}$ (dimensionless). The results are presented as a function of (**a**) depth range, (**b**) dip range, (**c**) structural domains, (**d**) filling minerals, and (**e**) by borehole. Vertical grey dashed lines indicate hydrostatic (${P}_{c}^{*}=0$) and lithostatic pressures (${P}_{c}^{*}=1$). Lower subplots indicate the number of fractures in each bin. Bin size is 0.1.

The data analysis presented in the Fig. 5 shows that no fracture is below the hydrostatic conditions (${P}_{c}^{*}=0$), meaning that no fracture can be critically stressed by the hydrostatic pressure only with the chosen failure envelope. Between ${P}_{c}^{*}=0$ and ${P}_{c}^{*}=1$, the openness is well fitted by an exponentially decreasing function (orange dashed line) and reaches a plateau for ${P}_{c}^{*}>1$ with a mean value of 0.17. When extrapolated to ${P}_{c}^{*}=0$, the exponential function predicts an openness equal to $1$, meaning that a fracture that would be critically stressed under hydrostatic conditions would necessarily be open. As for the analysis with ${\sigma }_{n}$, the observed trend is nearly the same when considering different depth ranges, orientations, or structural domains (Fig. 5a–c), which indicates that the pressure metric ${P}_{c}^{*}$ is relevant to describe the openness state of a fracture whatever its characteristics (with the notable exception of fracture-filling minerals, which will be discussed later). The calculations performed by borehole reveal a similar variability as for the analysis with ${\sigma }_{n}$, with an average standard deviation of 0.13 (vertical error bars; Fig. 5e). The correlation coefficient ${r}_{w}$ presents less correlation as for ${\sigma }_{n}$ (${r}_{w}=-0.53$) when considering the entire dataset. However, a similar correlation coefficient is measured when considering the data between ${P}_{c}^{*}=0$ and ${P}_{c}^{*}=1$ (${r}_{w}=- 0.62$; Supplementary Materials: Table 1). For the fractures with ${P}_{c}^{*}>1$, there is no correlation with the openness (${r}_{w}\sim 0$).

### Impact of fracture mineral fillings on openness

Openness depends on the fracture-filling minerals as shown in Figs. 2d, 3d and 5d for different stress metrics. In general, fractures with older minerals are less open than recent ones (Supplementary Materials: Fig. 9). Note that a fracture generally contains several minerals, so there is some overlap between the fracture families grouped by filling mineral.

The results shown in Fig. 5d confirm that the openness is approximately constant when the failure overpressure metric is greater than lithostatic conditions (${P}_{c}^{*}>1$), but the level of these plateaus depends on minerals ranging from 0.1 for adularia and oxidized walls to 0.3 for chlorite. Using several failure envelope parameters ($c$ and $\mu$) in the range determined by mechanical tests^39^, we have noted that the level of the plateau at ${P}_{c}^{*}>1$ does not change (Supplementary Materials: Fig. 10), indicating that the different plateaus observed among minerals cannot be explained by the mineral strength. We thereafter note ${f}_{op}^{\infty }$ the openness at ${P}_{c}^{*}>1$.

Below lithostatic conditions (${P}_{c}^{*}<1$), the trends depend on the minerals present. For all those with ${f}_{op}^{\infty }$ less than $\sim$ 0.15 (oxidized walls, adularia, quartz), the fraction of open fractures remains rather low and constant, whatever the failure overpressure metric ${P}_{c}^{*}$. In contrast, for minerals with ${f}_{op}^{\infty }$ larger than ~ 0.2 (chlorite, calcite, hematite), the openness increases exponentially when the failure overpressure decreases with a similar trend to that measured for all fractures. Laumontite-filled fractures, whose limit openness ${f}_{op}^{\infty }$ is around 0.2, shows an intermediate behaviour, with a slight increase in the openness when ${P}_{c}^{*}$ decreases.

**Figure 6:**
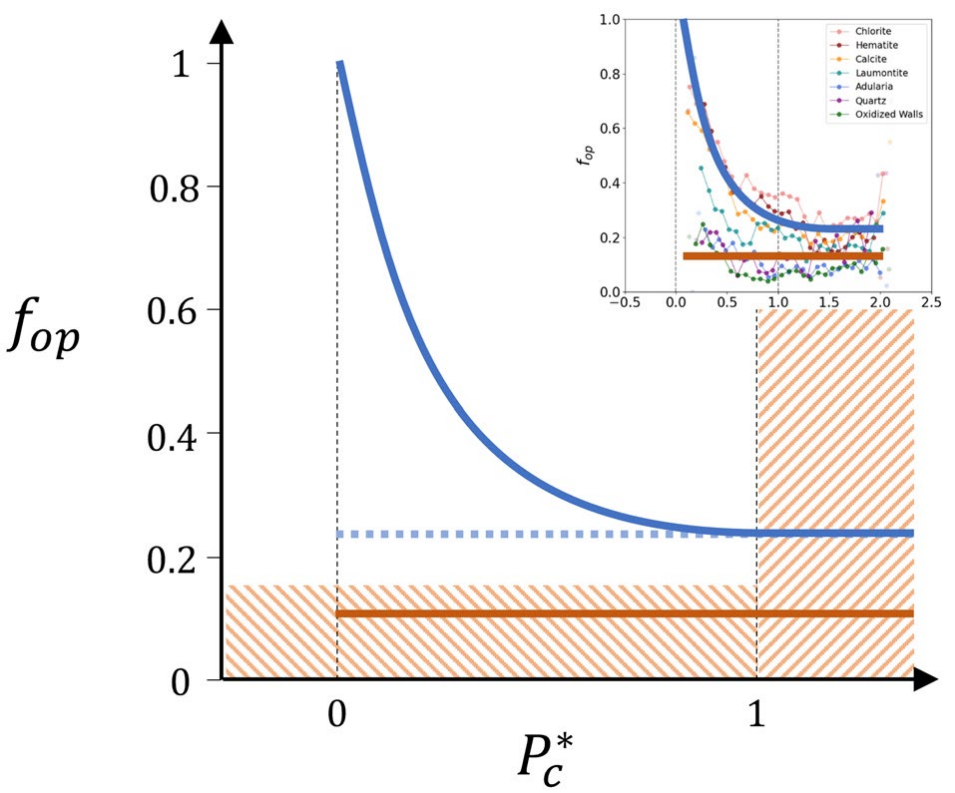
Schematic interpretation of the dependency of mineral fillings on fracture openness representing the openness as a function of the failure overpressure metrics ${P}_{c}^{*}$. The shaded areas (${P}_{c}^{*}>1$ and ${f}_{op}<0.15$) indicate fractures that cannot be reactivated (see text). The red and blue solid lines are indicative of the two main trends observed, and the blue dashed line sketches the openness of the blue group’s fractures before reactivation. The box at the top right shows the original data and interpretations.

Both trends described above are sketched in Fig. 6.

## Discussion

### Can overpressure control fracture openness?

Although it may be an overinterpretation, it is tempting to find a logic in these results. First, we note that since the fluid overpressure is not likely larger than the lithostatic conditions, no reactivation is possible with the current stress conditions for fractures with ${P}_{c}^{*}>1$. This implies that the openness of those fractures is inherited from previous conditions, probably the very early phases of mineralisation that took place in Forsmark. It is therefore not surprising that the fracture openness varies according to the fracture-filling minerals, and that for a given mineral, the fracture openness is independent of ${P}_{c}^{*}$ as long as ${P}_{c}^{*}>1$.

Because the metric ${P}_{c}^{*}$ is a distance to failure by overpressure, it is thus tempting to link the increase in openness with the ease of fracture reactivation, i.e., with the decrease of ${P}_{c}^{*}$ (blue solid line in Fig. 6). The exponential increase may reflect the probability of occurrence of failure by overpressures since a low occurrence is expected for strong overpressures. It may also reflect the reactivation dynamics where the pressure build-up is favoured by the low connectivity of fracture clusters, while fracture opening by reactivation tends to increase connectivity and thus to make the overpressure drop, inhibiting further reactivation.

The attempted explanation does not apply to fractures with low openness (red solid line in Fig. 6). The fact that the openness of those fractures is approximately constant regardless of the mechanical parameters (see also Figs. 2d, 3d, and 5d) indicates that reactivation is hardly possible for those fractures, either because of their low porosity, which limits the fracture area where fluid pressure can be applied, or because of the impossibility of building up high overpressures in the associated networks.

To summarise, the interpretation of the fracture openness data presented above could be:

1. Fractures had an initial openness inherited from the old mineralisation stages (blue dashed line and red solid lines).
2. The initial openness can be measured on the current network under conditions where reactivation is impossible (${P}_{c}^{*}>1$).
3. The mechanical reactivation under the current stress leads to an increase in openness with an exponentially decreasing efficiency as the overpressure required for failure increases or if the initial openness is small (i.e., smaller than ~ 0.15).

### Could past glacial loading and unloading cycles be the cause of the fracture reactivation?

The observation that the current state of stress might control the openness led us to question more deeply whether and how fractures may have been reactivated recently. At Forsmark, most of the fractures formed prior to 1 Ga, and reactivation occurred between 460 and 277 Ma along with mineral precipitation^52^. The possibility of further substantial reactivation during the quaternary glacial-interglacial cycles is an issue for the long-term safety of radioactive waste disposal sites in a future scenario of continental ice-sheet development^53^. Considering that, with few exceptions, open fractures are not critically stressed with the current stress field and the failure envelope parameters measured on Forsmark rocks, a recent reactivation of the fractures cannot be achieved without invoking fluid overpressures. The scenario of ice-sheet development has been modelled by several authors^24,27,46,47,54,55^. Critical situations occur near the ice front due to overpressures generated below the warm-based ice-sheet, or the development of an impermeable permafrost layer, entailing both possible jacking and fracture shearing^24–26^. The potential for overpressures at depth depends on a large number of unconstrained parameters: pressure diffusivity (ratio of permeability and water volume), glacier geometry, advance/retreat speed (because of transient pressure diffusion). Depending on the model, the ice-sheet causes overpressures of 2–6 MPa at 200–300 m, the maximum jacking depth^24,47^. At these depths, this corresponds to a failure overpressure metric ${P}_{c}^{*}$ between ~ 0.1 and ~ 0.5 (Supplementary Materials: Fig. 4). But all these models oversimplify the fracture structure observed at Forsmark, where a wide range of fracture sizes and transmissivities leads to a complex network of more or less connected fracture clusters^36,56–58^. The conditions of pressure build-up and reactivation in these complex systems during glacial-interglacial cycles have yet to be addressed.

## Conclusion

The objectives of the present paper were to assess how much the openness relates to the current stress field and evaluate which fracture reactivation mechanisms are involved in Forsmark. In the continuity of the study of Moon et al.^12^, we studied correlations between the open fraction ${f}_{op}$ and different stress indicators: the normal stress ${\sigma }_{n}$, likely reflecting tensile reactivation; the distance to shear failure $\tau /{\tau }_{c}$; and finally, the potential pressure required for failure ${P}_{c}$, reflecting failure assisted by fluid pressure. The results showed that ${\sigma }_{n}$ has an important control on fracture reactivation, whatever fracture depth and orientation, even at large depths (until 1000 m deep). On the contrary, the correlation with $\tau /{\tau }_{c}$ was weaker and varied with fracture orientation and depth. When analysing the pressure indicator ${P}_{c}$, significant correlations were measured for ${P}_{c}$ between hydrostatic and lithostatic pressures, which let us suggest a possible impact of fluid overpressure in the process of fracture reactivation.

## Methods

### Dataset description

The cored boreholes consist mainly of a series of shallow and deep boreholes (*KFM01–KFM12*) drilled between 2002 and 2006 and distributed across the area of the future repository site (Supplementary Materials). We also include two series of shallow boreholes, drilled in 2005 (*KFM90A-F*) and between 2011 and 2016 (*KFM13–KFM23*). The latter series are in the north-west of the area, near the nuclear power station. Along with core mapping, borehole imaging using BIPS (Borehole Image Processing System) was also performed and interpreted in these boreholes. The results of the interpretations are available in the SKB database *SICADA*. Our dataset is composed of the following fracture information: fracture position, orientation, open/partly-open/sealed interpretation, and mineral and wall alteration types. To distinguish open fractures due to the drilling from natural open fracture, fractures were first qualified as broken or unbroken directly from the core observations. Broken fractures are referred to as open if a distinguishable aperture is observable in the borehole image, if the fracture surfaces are weathered or altered, or if the fracture walls do not match. Otherwise, they are classified as sealed. Unbroken fractures are classified as sealed unless an aperture is distinguishable, in which case it is classified as partly open (SKB^59^—see 1.6.5, Nomenclature). In this study, partly open fractures (2% of the dataset) are classified as open.

### Openness calculation

The core-log data provide one-directional information with an apparent fracture intensity (${m}^{-1}$). A well-known assumption is that this intensity statistically quantifies the total fracture surface per unit volume, called the density ${p}_{32}$ (${m}^{2}.{m}^{-3}$), when an angular correction is applied to the fracture^60,61^. This angular correction avoids undersampling due to the fracture orientation relative to the borehole direction. Similarly, we assume that the proportion of open fracture measured in the core-log data can provide, with the angular correction, an estimate of the open fracture surface fraction in the fracture network, which we call openness. Note that we choose to analyse the proportion of open fracture surface independent of the underlying fracture density. This latter may result from old deformation episodes, decorrelated from the present stress state.

In any subset of fracture, for example, when a binning is performed relative to a fracture parameter (here ${\sigma }_{n}$, $\tau$, and ${P}_{c}$), the openness, noted ${f}_{op}$, is calculated as:

1 $${f}_{op}=\frac{{\sum }_{f\in [{F}_{op}]}{C}_{f}}{{\sum }_{f\in [F]}{C}_{f}}$$

$[F]$ is a selected set of fractures and [${F}_{op}]$ is the subset of $[F]$ that is classified as open. ${C}_{f}$ is the angular correction of fracture $f$, and is calculated as ${C}_{f}= \frac{1}{\mathrm{sin}\left({\alpha }_{f}\right)}$, with ${\alpha }_{f}$ the acute angle between the fracture $f$ and the borehole direction, deduced from the trace. To avoid infinite correction values when fractures are quasi-parallel to the borehole, we assume that ${\alpha }_{f}$ cannot be less than $5^\circ$, which accounts for orientation uncertainty measurement.

### Normal stress ${{\varvec{\sigma}}}_{{\varvec{n}}}$ and shear stress ${\varvec{\tau}}$ in the fracture

The fracture’s normal stress ${\sigma }_{n}$ and shear stress $\tau$ are calculated from the site scale stress model of Forsmark^38^. The stress state is characterised by three principal components evolving with depth, and with a minimum vertical component and horizontal intermediate and maximal components (Supplementary Materials). All stress components are compressive. In this study, we adopt the sign convention in which compressive stresses are positive.

The stress vector applied on the fracture plane $\overrightarrow{t}$ is defined as:

2 $$\overrightarrow{t}=\overline{\overline{T}}(d).\overrightarrow{n}$$

with $\overline{\overline{T}}(d)$ the stress tensor that depends on fracture depth $d$, and $\overrightarrow{n}$ the normal vector to the fracture. The fracture normal stress is calculated as:

3 $${\sigma }_{n}=\overrightarrow{n}(\overrightarrow{t}.\overrightarrow{n})$$

and shear stress as^62^:

4 $$\tau =\overrightarrow{n}\times (\overrightarrow{t}\times \overrightarrow{n})$$

### Fluid pressure analyses

The theoretical fluid pressure ${P}_{c}$ necessary for the fracture to reach the failure envelope is calculated as:

5 $${P}_{c}={\sigma }_{n}-\frac{\tau -c}{\mu }$$

where ${\sigma }_{n}$ and $\tau$ are the fracture’s normal and shear stress, and $c$ and $\mu$ the cohesion and friction of the Mohr–Coulomb failure envelope.

To take account of the variation of overpressure with depth, we normalise it by both the hydrostatic pressure ${P}_{H}$ (${P}_{H}={\gamma }_{w}z$ with ${\gamma }_{w}$= 9.81 $\times$ 10^–3^ MPa/m) and the lithostatic pressure ${P}_{L}$:

6 $${P}_{c}^{*}=\frac{{P}_{c}- {P}_{H}}{{P}_{L}- {P}_{H}}$$

The lithostatic pressure is calculated as the average of the three principal stress components of the Forsmark stress model,

7 $${P}_{L}=\frac{{\sigma }_{H}+{\sigma }_{h}+{\sigma }_{v}}{3}$$

This formulation is used because the dataset is relatively close to the surface. We choose to not neglect the deviatoric stresses ($\left|{\sigma }_{i}-{\sigma }_{j}\right|$), as is usually done in other studies that consider the lithostatic stress as the overburden stress, ${\sigma }_{v}$ only^22,49,50^.

### Weighted Pearson correlation coefficient ${{\varvec{r}}}_{{\varvec{w}}}$

We evaluate the correlations between the openness ${f}_{op}$ and the stress indicators with the Pearson correlation coefficient weighted by the fracture number in each bin. It is calculated as follows, considering two variables $x$ and $y$, and a weight vector $w$:

8 $${r}_{w} \left(x,y,w\right)=\frac{cov\left(x,y,w\right)}{\sqrt{cov\left(x,x,w\right).cov(y,y,w)}}$$

with $cov\left(x,y,w\right)$ the weighted covariance defined as:

9 $$cov\left(x,y,w\right)=\frac{\sum_{i}\left({w}_{i}\left({x}_{i}-m\left(x,w\right)\right)\left({y}_{i}-m\left(y,w\right)\right)\right)}{\sum_{i}{w}_{i}}$$

$m\left(x,w\right)$ is the weighted mean:

10 $$m\left(x,w\right)=\frac{\sum_{i}{w}_{i}{x}_{i}}{\sum_{i}{w}_{i}}$$

The indicator ${r}_{w}$ varies between -1 and 1. If close to 0, there is no correlation between the two variables. If close to 1, there is a positive correlation. If close to − 1, there is a negative correlation.

## Supplementary Information

Supplementary Information.

## Data Availability

The dataset analysed during the current study is available in the following repository:  https://github.com/didoolaeghe/Forsmark-Openness-Analysis.

## Code availability

The analysis codes used during the current study are available in the following repository:  https://github.com/didoolaeghe/Forsmark-Openness-Analysis.
