# Supplementary material for: Controls on fracture openness and reactivation in Forsmark, Sweden
Source: Sci Rep. 2023 Apr 24;13:6686. doi: 10.1038/s41598-023-33619-9 (PMC10126104; doi:10.1038/s41598-023-33619-9)
Supplement: Supplementary file 1 — Supplementary Information. [file 41598_2023_33619_MOESM1_ESM.docx]

# Supplementary Materials


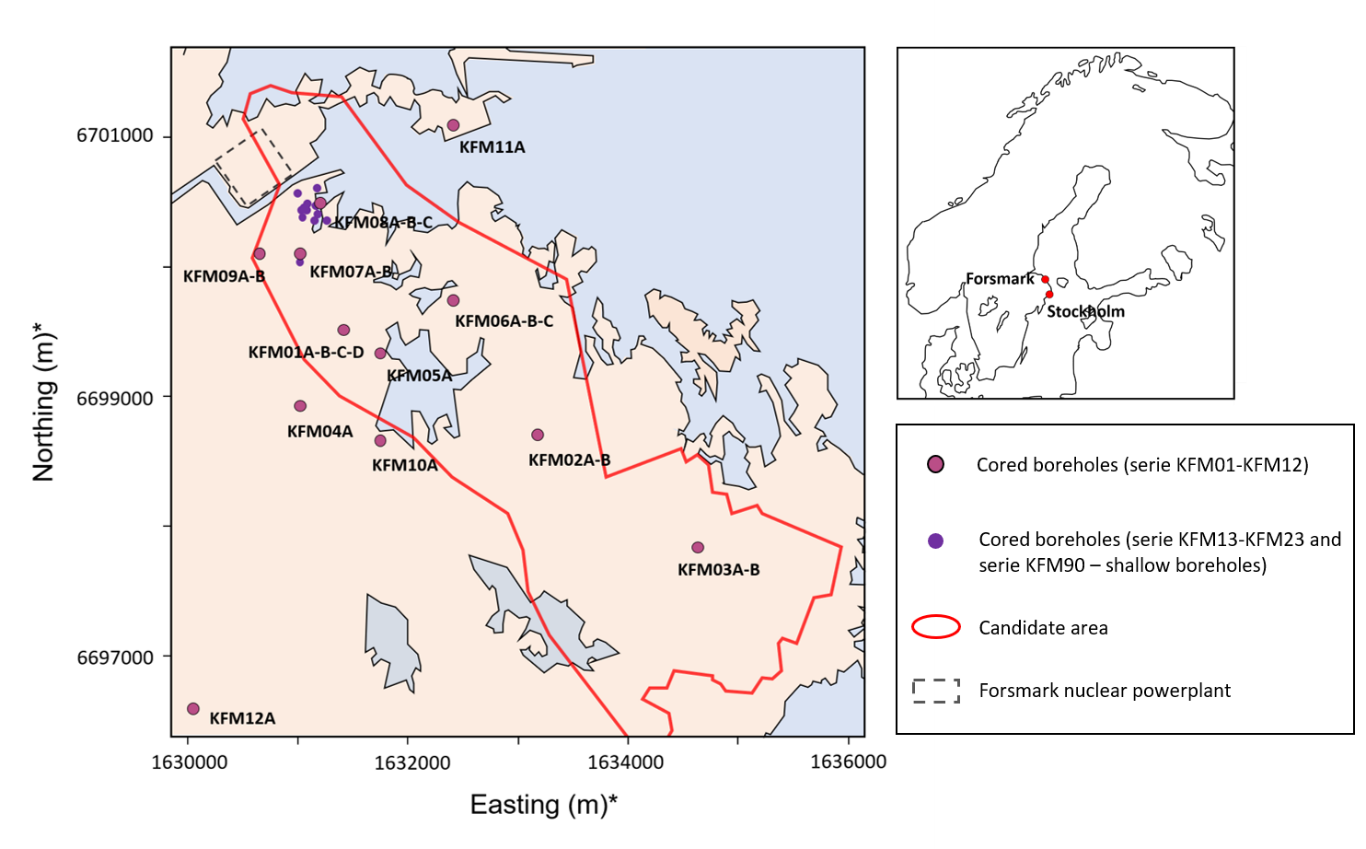


**Supplementary figure 1 : Positions of the cored borehole tops at the Forsmark site.** These positions are plotted from the SICADA database. The other elements, i.e., coastline, candidate area, and power plant, are plotted using a combination of figures from the report R-07-45 ^1^. Courtesy of SKB. * Easting and northing are measured in the RT90 coordinate system.


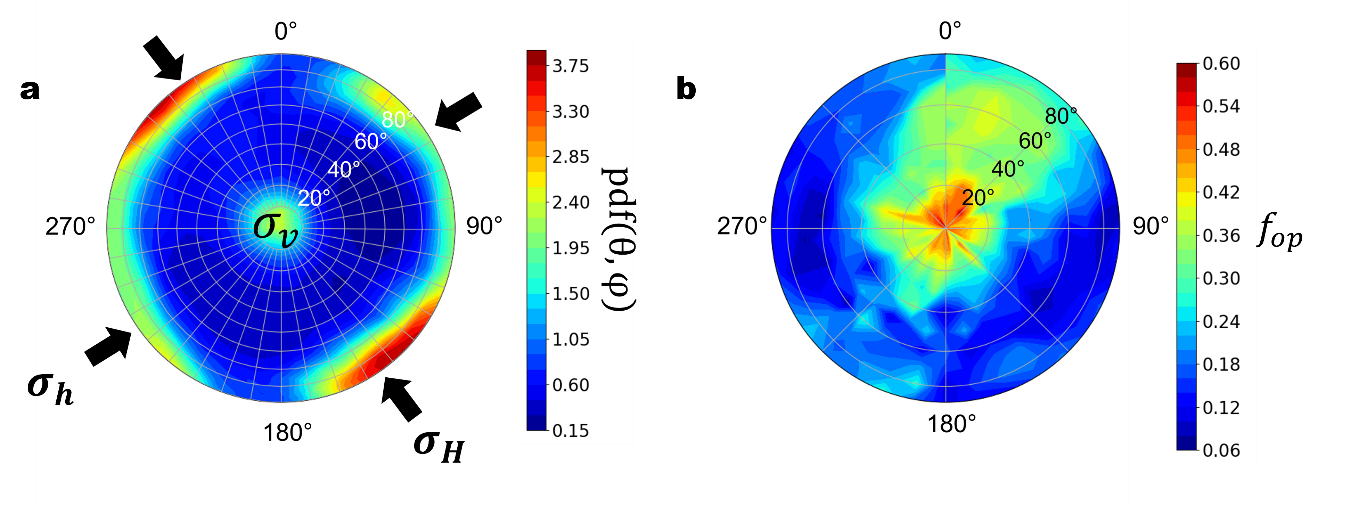


**Supplementary figure 2 : Stereonet representations of fractures and openness orientations.** (a) Classical stereonet of all fractures. The density colour scale represents the fracture density distribution according to orientation units, $pdf(\theta,\varphi$). (b) Openness as a function of the fracture orientation. In (a) and (b), as per the stereonet plot convention, the fracture pole downward direction is read around the polar grid, with north being up (0°), and the fracture dip is indicated from the middle (0°) to the edge of the stereonet (90°). We recall that in (a) and (b), the fractures are weighted with a Terzaghi correction. In (a), $\sigma_{v}, \sigma_{h}$and $\sigma_{H}$ indicate the directions of the three principal components of the Forsmark stress model ^2^.


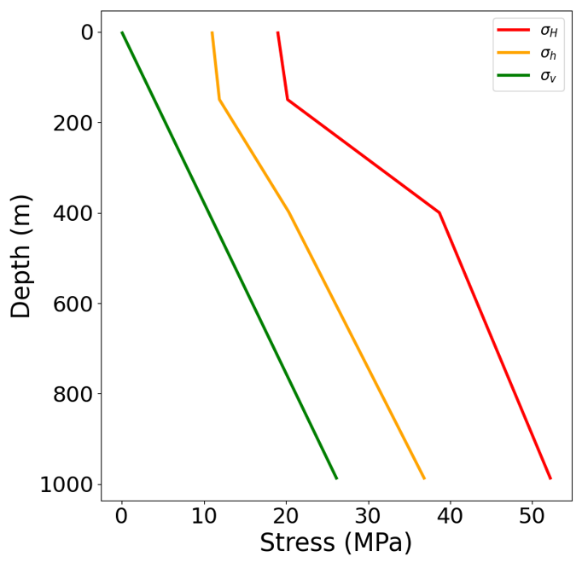


**Supplementary figure 3 : Magnitudes of the principal components of Forsmark stress model, as a function of depth.** The maximum and intermediate components, $\sigma_{H}$ and $\sigma_{h}$, are horizontal, and the minor component, $\sigma_{v},$ is vertical ^2^. Depth is measured relative to sea level.


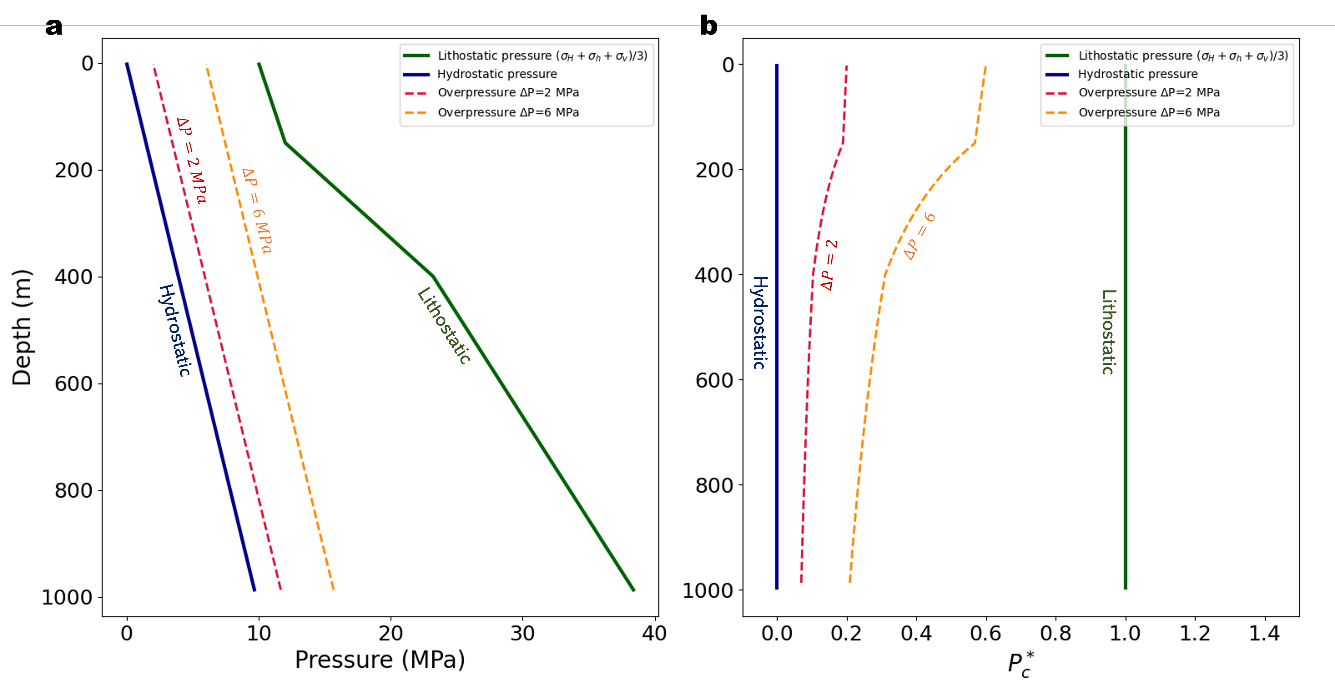


**Supplementary figure 4: Pressures and overpressures as a function of depth.** (a) Hydrostatic and lithostatic pressure as a function of depth. Overpressures $\Delta P$of 2 and 6 MPa above the hydrostatic pressure are represented, and correspond to simulated ice-sheet overpressures at depths of 200-300 meters ^3,4^. (b) Same quantities converted in our failure overpressure metric $P_{c}^{*}$.


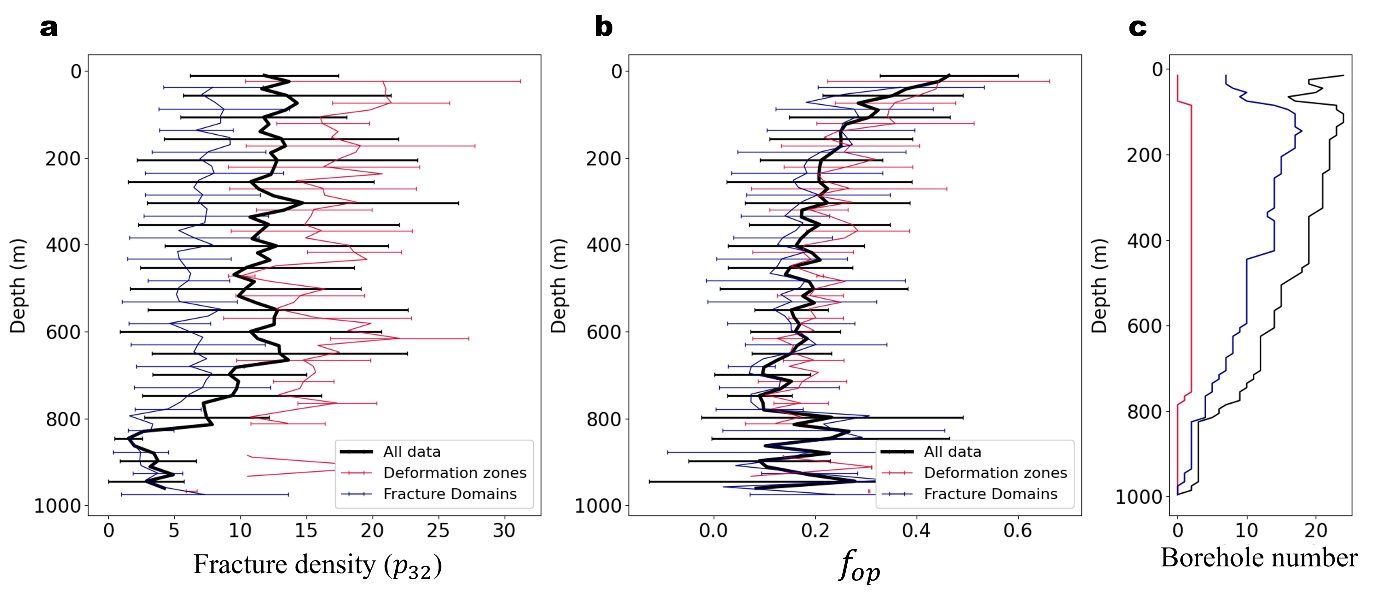


**Supplementary figure 5: Density and openness as a function of depth.** (a) Average fracture density ($p_{32}$) as a function of depth, (b) average openness as a function of depth, (c) number of boreholes at a given depth. The measures are performed for all fractures (black), fractures in deformation zones (red), and fractures in fracture domains (blue). Horizontal error bars indicate the standard deviation of measurements by boreholes. Depth is measured relative to sea level.


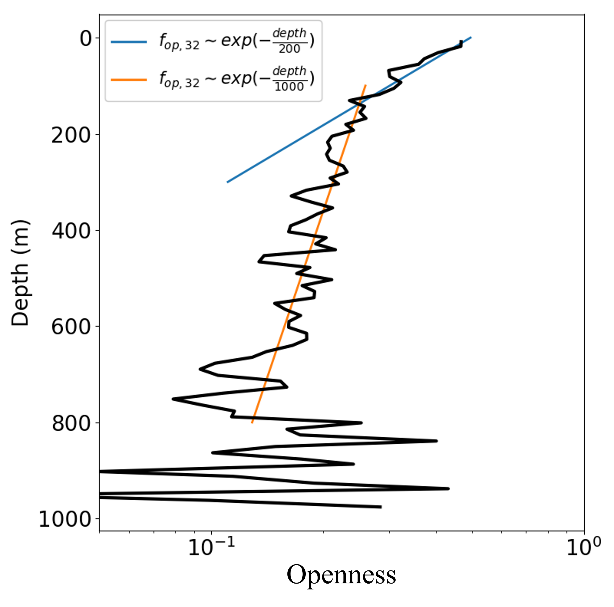


**Supplementary figure 6 : Exponential fits on the average openness as a function of depth.**


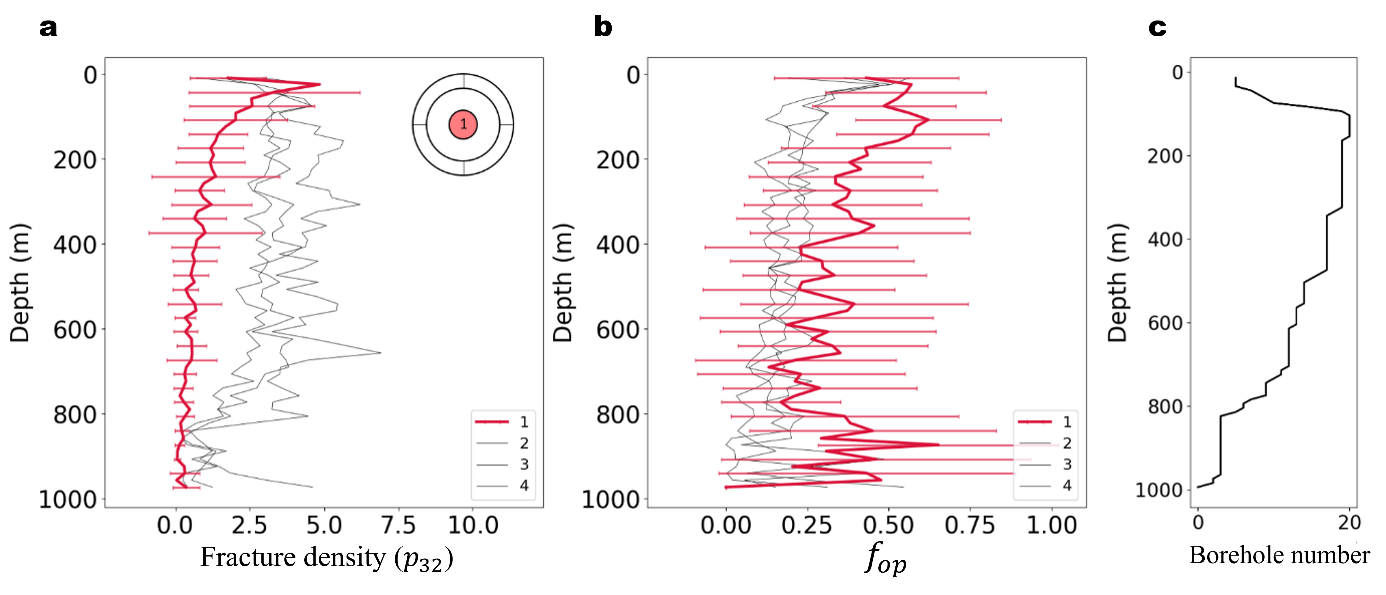


**Supplementary figure 7:** **Density and openness as a function of depth in horizontal fractures.** (a) Density ($p_{32}$). (b) Openness. (c) Number of boreholes at a given depth. The horizontal group is defined as: dip $\theta<20^{\circ}$. Horizontal error bars indicate the standard deviation of measurements by boreholes. Depth is measured relative to sea level.


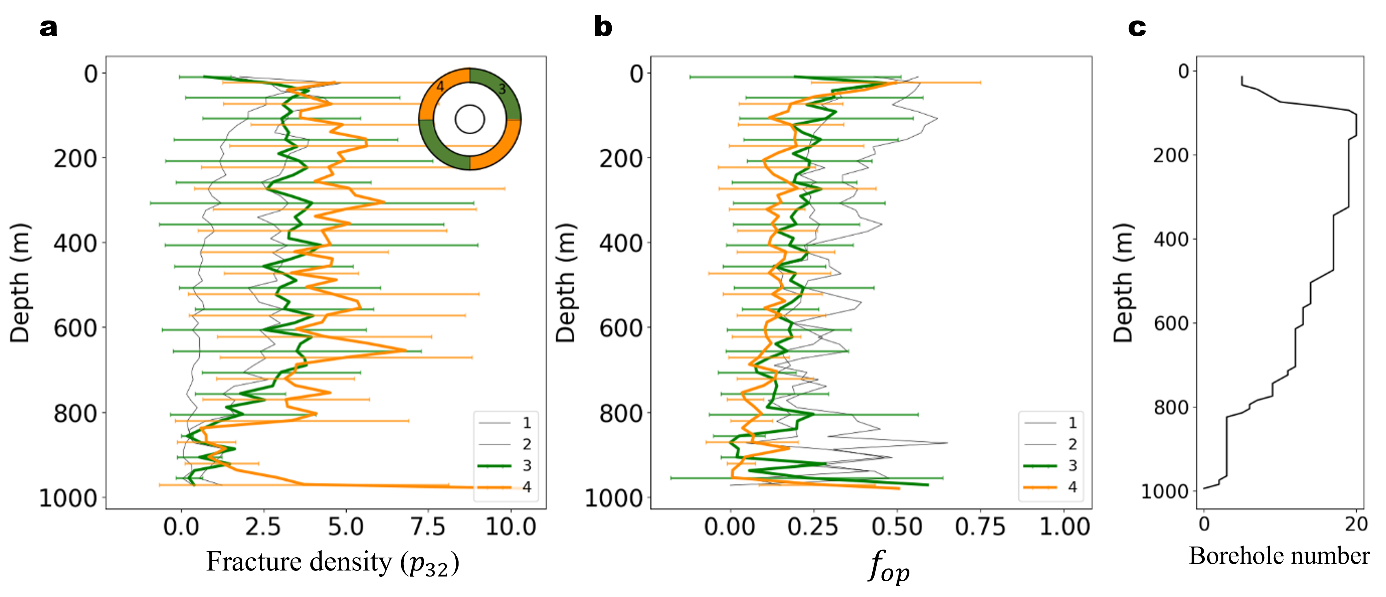


**Supplementary figure 8:** **Density and openness as a function of depth in vertical fractures.** (a) Density ($p_{32}$). (b) Openness. (c) Number of boreholes at a given depth. In the first vertical set (green lines), dip $\theta>65^{\circ}$ and pole direction $\varphi\epsilon\left( \left[ 0^{\circ},90^{\circ} \right]\cup\left[ 180^{\circ},270^{\circ} \right] \right)$. In the second vertical set (yellow lines), dip $\theta>65^{\circ}$ and pole direction $\varphi\epsilon\left( \left[ 90^{\circ},180^{\circ} \right]\cup\left[ 270^{\circ},360^{\circ} \right] \right)$. Horizontal error bars indicate the standard deviation of measurements by boreholes. Depth is measured relative to sea level.


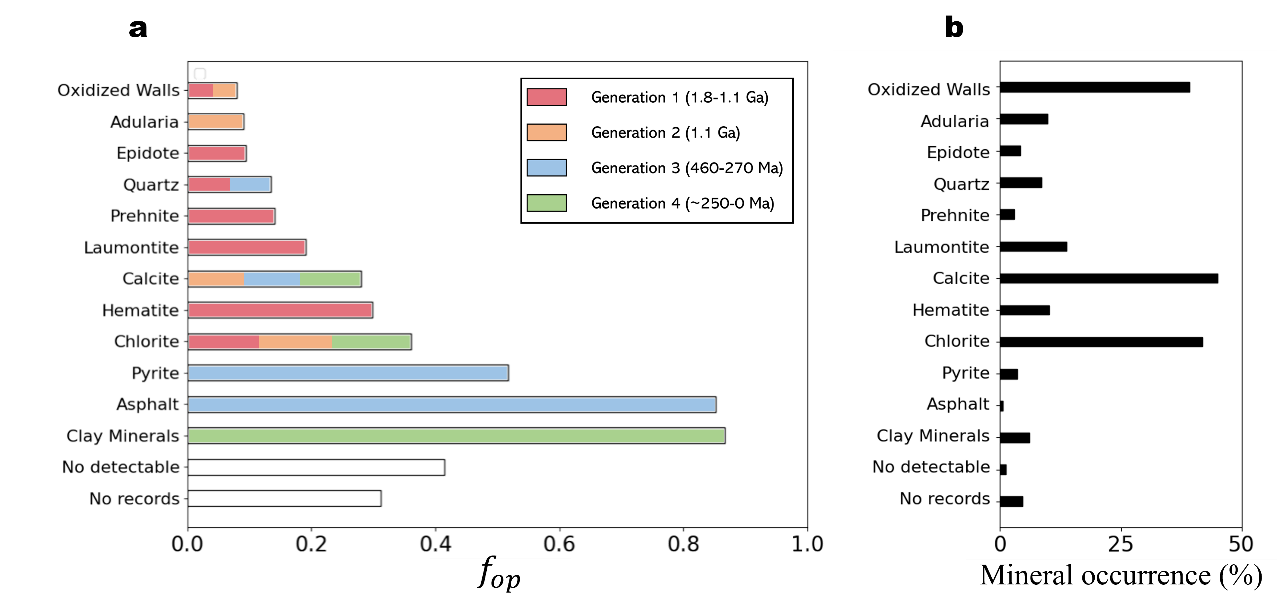


**Supplementary figure 9 : Openness according to mineral fillings.** (a) The openness is computed in groups of fractures selected by the presence of a mineral in the fracture. Colours indicate the generations the mineral belongs to ^5^. (b) Mineral occurrence in percentage.

**
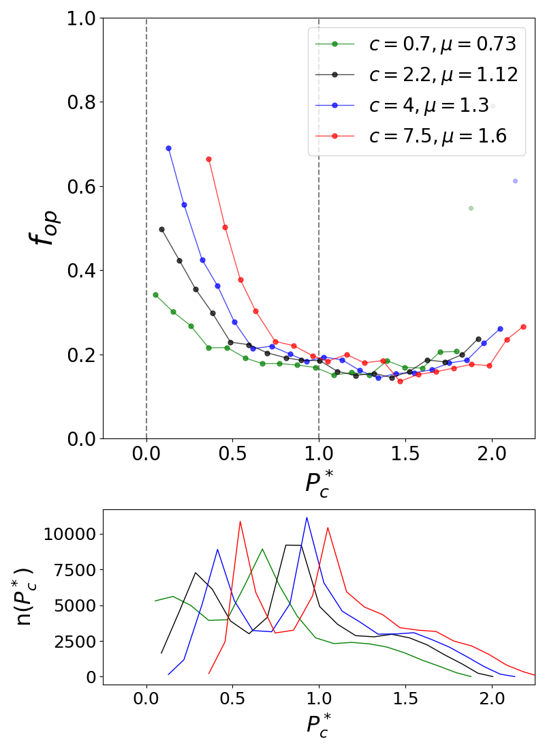
**

**Supplementary figure 10: Openness as a function of normalised critical pressure** $\boldsymbol{P}_{\boldsymbol{c}}^{\boldsymbol{*}}$**, using different failure envelope parameters,** $\boldsymbol{c}$ **and** $\boldsymbol{\mu}$**.** The different failure envelopes are from measurements in Glamheden, et al. ^6^. Green: measurements on open fractures. Black, blue, and red: minimum, average, and maximum values, respectively, for measurements on sealed fractures. The notation $P_{c}^{*}$means that the fluid pressure is normalised by both the hydrostatic and lithostatic pressures (see Method). Lower subplots indicate the number of fractures in each bin. Bin size is 0.1.


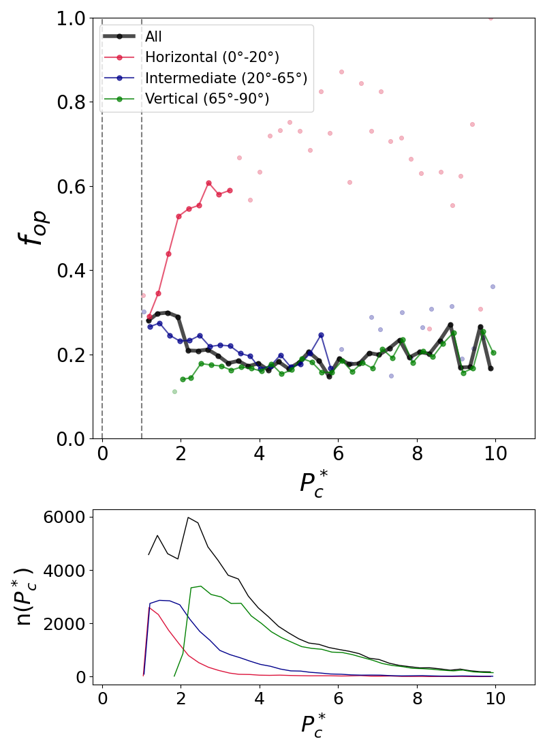


**Supplementary figure 11 : Openness as a function of normalised critical pressure** $\boldsymbol{P}_{\boldsymbol{c}}^{\boldsymbol{*}}$**, with lithostatic pressure defined as the overburden** $\boldsymbol{\sigma}_{\boldsymbol{v}}\boldsymbol{.}$The notation $P_{c}^{*}$means that the fluid pressure is normalised by both the hydrostatic and lithostatic pressures (see Method). Vertical grey dashed lines indicate hydrostatic ($P_{c}^{*}=0$) and lithostatic pressures ($P_{c}^{*}=1$). Transparent dots correspond to bins with fewer than 100 fractures. Lower subplots indicate the number of fractures in each bin. Bin size is 0.25.


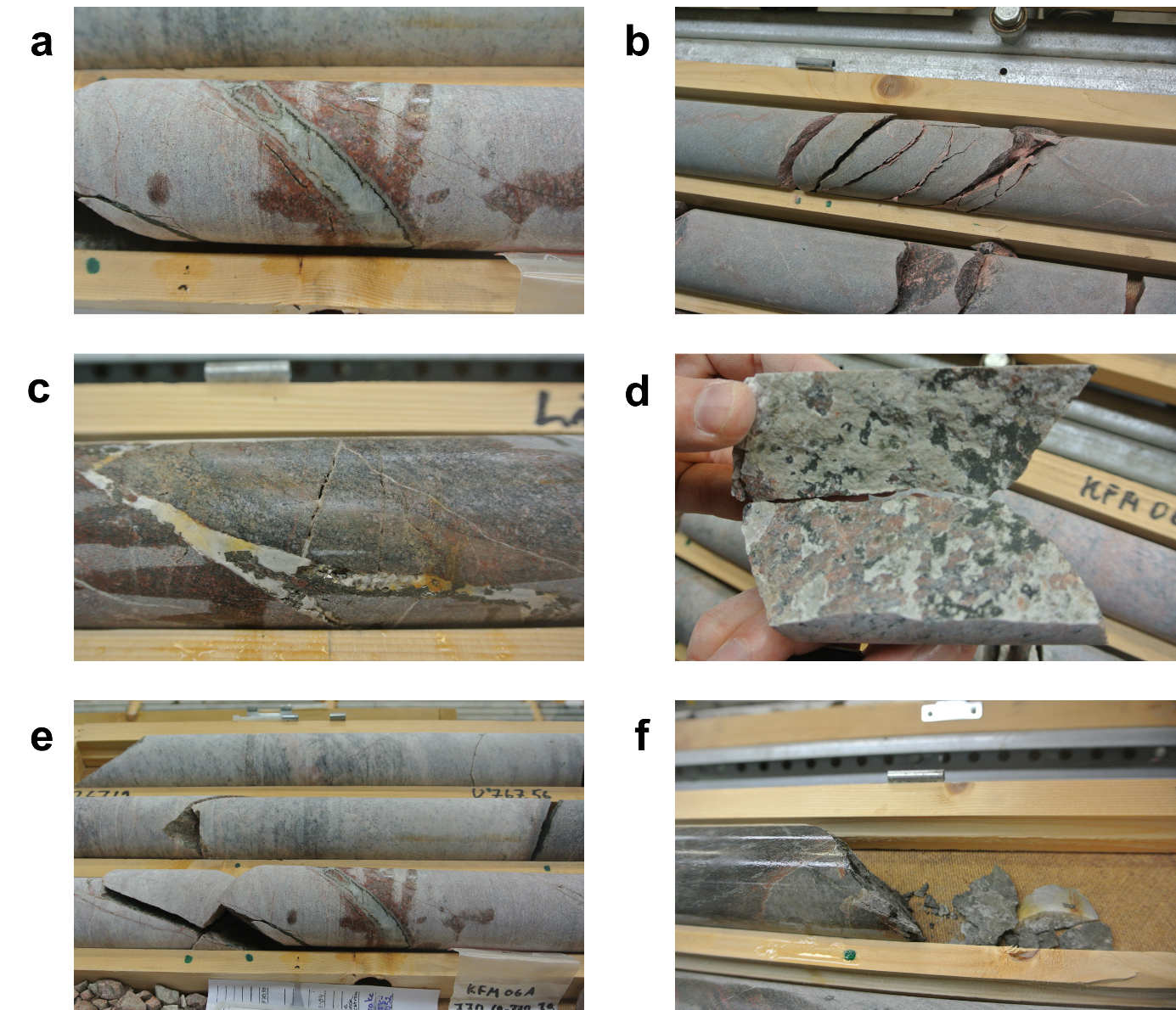


**Supplementary figure 12: Example of photographs of Forsmark’s cores.** Courtesy of Diane Doolaeghe. (a) Fracture sealed with prehnite, chlorite, and calcite ($\sim$-770m, in borehole KFM06A). (b) Broken fractures filled with Laumontite ($\sim$-459m, in borehole KFM11A). (c) Sealed and partly open fractures with calcite precipitated together with pyrite ($\sim$-454m, in borehole KFM11A). (d) Broken fracture with thin coatings of calcite and clay minerals ($\sim$-132m, in borehole KFM06A). (e) Examples of open and sealed fractures ($\sim$-750m, KFM06A). (f) Open fracture recorded as flowing, with clay sealing ($\sim$-470m, KFM11A).


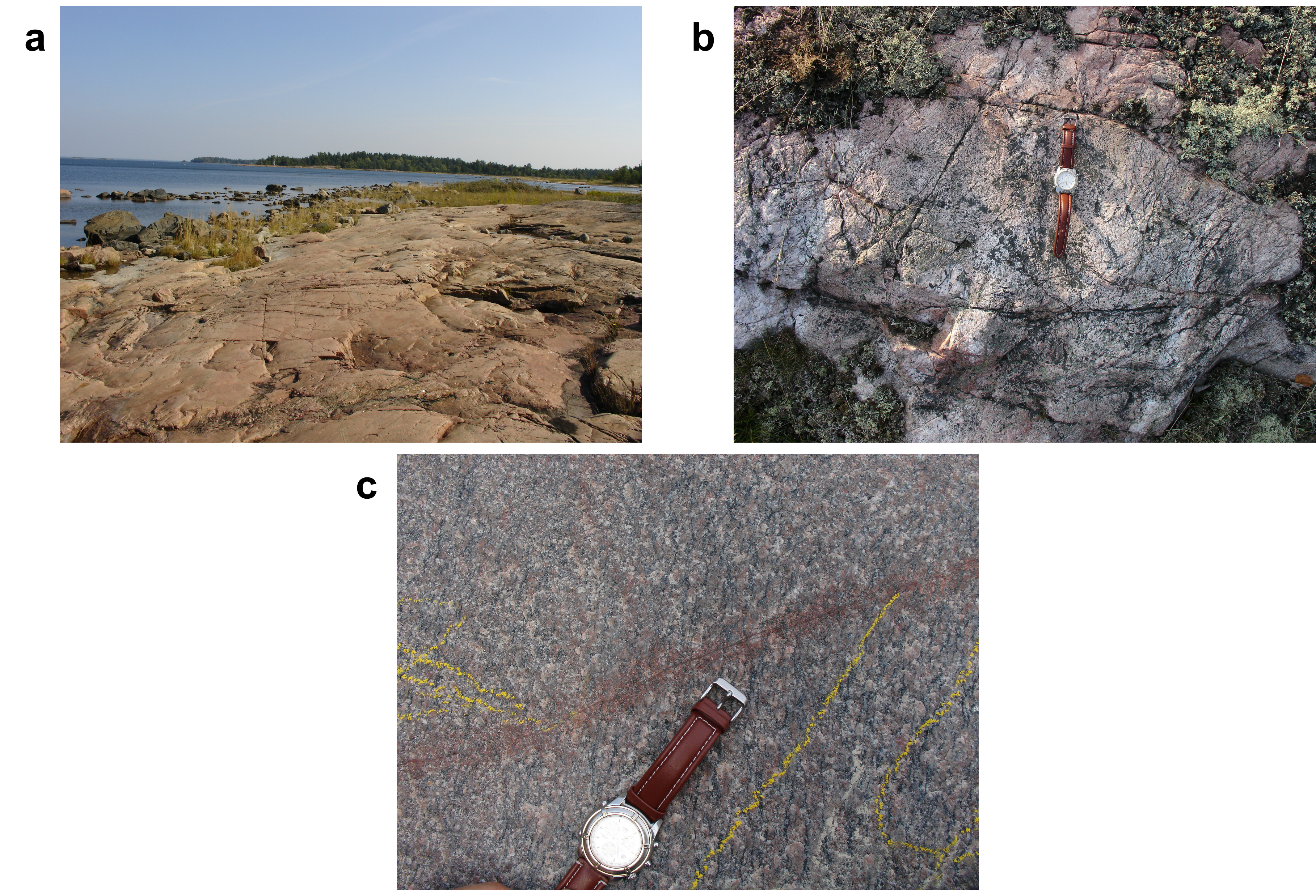


**Supplementary figure 13: Photographs of fractures on surface outcrops in Forsmark.** Courtesy of Philippe Davy.

|  | | Weighted Pearson correlation coefficient $r_{w}$ | Spearman ranking coefficient |
| --- | --- | --- | --- |
| $\sigma_{n}$ | All | -0.62 | -0.67 |
|  | Under 10 MPa | -0.61 | -0.55 |
|  | Above 10 MPa | -0.47 | -0.46 |
| $\frac{\tau}{\tau_{c}}$ | All | 0.49 | 0.49 |
| $P_{c}^{*}$ | All | -0.53 | -0.49 |
|  | Under 1 | -0.62 | -0.64 |
|  | Under 1 (exponential) | -0.62 | -0.66 |
|  | Above 1 | 0.05 | -0.08 |

**Supplementary table 1: Correlation coefficients between the openness** $\boldsymbol{f}_{\boldsymbol{op}}$ **calculated by borehole and the stress indicators,** $\boldsymbol{\sigma}_{\boldsymbol{n}}$**,** $\boldsymbol{\tau/}\boldsymbol{\tau}_{\boldsymbol{c}}$**, and** $\boldsymbol{P}_{\boldsymbol{c}}^{\boldsymbol{*}}$ (see Figures 2e, 3e, and 5e)**.** For the normal stress $\sigma_{n}$, the correlation is measured for all data and for data under and above 10 MPa, because we observed two different linear regimes under and above this value. For the fluid pressure indicator $P_{c}^{*}$, the correlation is measured for all data and for data under and above $P_{c}^{*}=1$. The Pearson correlation coefficient measures linear correlations (see Method 5). For $P_{c}^{*}<1$, we also calculated the correlation between $P_{c}^{*}$ and $ln(f_{op})$ ($ln$ is the natural logarithm) to evaluate the exponential relationship that we observed (Figure 5). The Spearman ranking coefficient measures correlations between the rank of the values. It assesses how well the relationship between two variables can be described by a monotonic function.

1 Stephens, M. B. *et al.* Geology forsmark. site descriptive modelling forsmark-stage 2.2. Report No. R-07-45, (Swedish Nuclear Fuel and Waste Management Co., Stockholm, Sweden, 2007).

2 Martin, C. D. Quantifying in situ stress magnitudes and orientations for Forsmark. Forsmark stage 2.2. Report No. R-07-26, (Swedish Nuclear Fuel and Waste Management Co., Stockholm, Sweden, 2007).

3 Selvadurai, A. P. S., Suvorov, A. P. & Selvadurai, P. A. Thermo-hydro-mechanical processes in fractured rock formations during a glacial advance. *Geosci. Model Dev.* **8**, 2167-2185, doi:<http://doi.org/10.5194/gmd-8-2167-2015> (2015).

4 Lönnqvist, M. & Hökmark, H. Approach to estimating the maximum depth for glacially induced hydraulic jacking in fractured crystalline rock at Forsmark, Sweden. *Journal of Geophysical Research: Earth Surface* **118**, 1777-1791, doi:<https://doi.org/10.1002/jgrf.20106> (2013).

5 Sandström, B. *Fluid Migration and Brittle Tectonothermal Evolution in the Central Fennoscandian Shield-Recorded by Fracture Minerals and Wall Rock Alteration*, University of Gothenburg, Department of Earth Sciences; Institutionen för geovetenskaper, (2009).

6 Glamheden, R. *et al.* in *SKB reports* Vol. R-07-15 (Swedish Nuclear Fuel and Waste Management Co., 2007).
